# Azithromycin combination therapy for community-acquired pneumonia: propensity score analysis

**DOI:** 10.1038/s41598-019-54922-4

**Published:** 2019-12-05

**Authors:** Akihiro Ito, Tadashi Ishida, Hiromasa Tachibana, Hironobu Tokumasu, Akio Yamazaki, Yasuyoshi Washio

**Affiliations:** 1 Department of Respiratory Medicine, Ohara Healthcare Foundation, Kurashiki Central Hospital, Miwa 1-1-1, Kurashiki, Okayama 710-8602 Japan; 2 Department of Respiratory Medicine, National Hospital Organization Minami Kyoto Hospital, Nakaashihara 11, Joyo, Kyoto 610-0113 Japan; 3 Department of Clinical Research Institute, Ohara Healthcare Foundation, Kurashiki Central Hospital, Miwa 1-1-1, Kurashiki, Okayama 710-8602 Japan; 4 Department of Respiratory Medicine, Shiga University of Medical Science Hospital, Tsukinowa Seta-cho, Otsu, Shiga 520-2192 Japan; 5 Research Institute for Diseases of the Chest, Graduate School of Medical Sciences, Kyushu University, Higashiku Maidashi 3-1-1, Fukuoka, Fukuoka 812-8582 Japan

**Keywords:** Antibiotics, Bacterial infection, Antimicrobial therapy

## Abstract

Whether macrolide combination therapy reduces the mortality of patients with severe community-acquired pneumonia (CAP) hospitalized in the non-intensive care unit (ICU) remains unclear. Therefore, we investigated the efficacy of adding azithromycin to β-lactam antibiotics for such patients. This prospective cohort study enrolled consecutive patients with CAP hospitalized in the non-ICU between October 2010 and November 2016. The 30-day mortality between β-lactam and azithromycin combination therapy and β-lactam monotherapy was compared in patients classified as mild to moderate and severe according to the CURB-65, Pneumonia Severity Index (PSI), and Infectious Diseases Society of America (IDSA)/American Thoracic Society (ATS) criteria. Inverse probability of treatment weighting (IPTW) analysis was used to reduce biases. Based on the CURB-65 and PSI, combination therapy did not significantly reduce the 30-day mortality in either group (179 patients in the combination group, 952 in the monotherapy group). However, based on the IDSA/ATS criteria, combination therapy significantly reduced the 30-day mortality in patients with severe (odds ratio [OR] 0.12, 95% confidence interval [CI] 0.007–0.57), but not non-severe pneumonia (OR 1.85, 95% CI 0.51–5.40); these results were similar after IPTW analysis. Azithromycin combination therapy significantly reduced the mortality of patients with severe CAP who met the IDSA/ATS criteria.

## Introduction

Among the infectious diseases, community-acquired pneumonia (CAP) is a major cause of hospitalization and death worldwide^1,2^. The cornerstone of CAP therapy is antibiotic agents, but the recommended treatment strategy differs among the guidelines^1,3,4^. Several previous retrospective and prospective observational studies have reported that β-lactam and macrolide combination therapy significantly improved the prognosis of hospitalized CAP patients^5–11^. However, two randomized controlled trials (RCTs) showed that combination therapy with β-lactam and macrolide did not significantly reduce the mortality of CAP patients hospitalized in the non-intensive care unit (ICU)^12,13^. Therefore, the benefit of adding macrolides to β-lactam antibiotics in improving the prognosis of CAP patients hospitalized in the non-ICU ward is controversial. A recent systematic review and meta-analysis reported that compared with β-lactam monotherapy, macrolide combination therapy reduced the mortality in severe pneumonia^14^. This result suggested the efficacy of β-lactam and macrolide combination therapy for severe CAP patients hospitalized in the non-ICU ward; however, no RCT has been conducted to test this hypothesis. Most of the studies, including two RCTs, used erythromycin, clarithromycin, and azithromycin as macrolides, but no study has reported the usefulness of azithromycin alone as the macrolide for combination therapy. Therefore, the aim of the present study was to assess the usefulness of azithromycin as the add-on macrolide to β-lactam antibiotics in patients hospitalized in the non-ICU ward for CAP, specifically the severe type, as defined by the CURB-65^15^, Pneumonia Severity Index (PSI)^16^, and Infectious Diseases Society of America (IDSA)/American Thoracic Society (ATS) severe pneumonia criteria^1^.

## Results

### Patients’ characteristics

**Figure 1:**
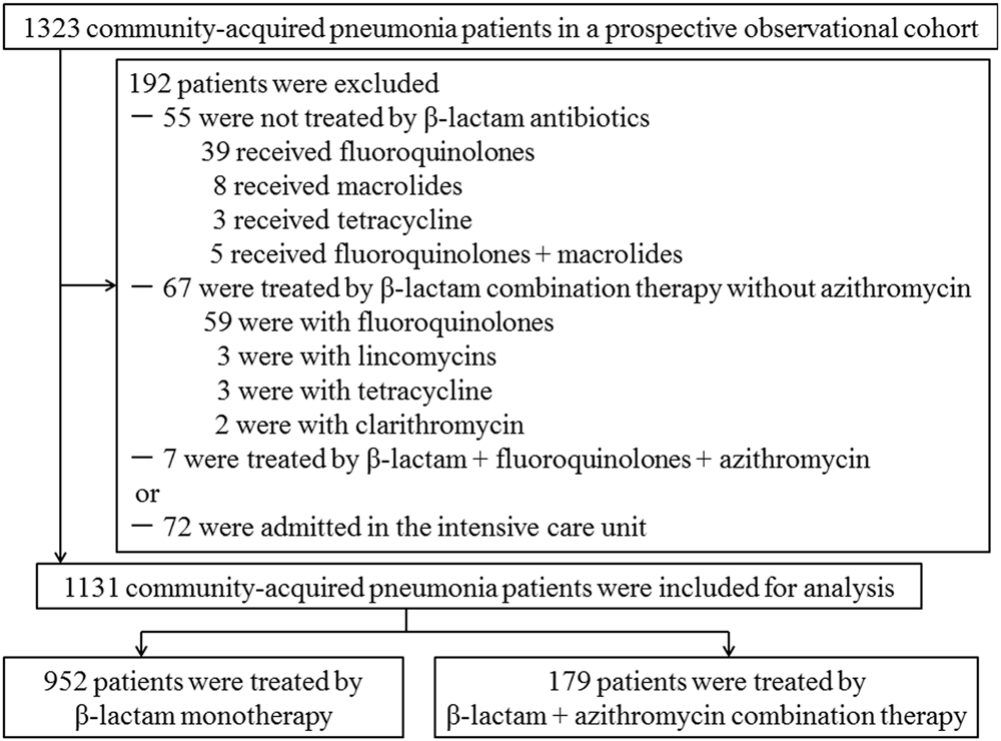
Study flowchart.

The flowchart of patient inclusion is shown in Fig. 1. A total of 1131 patients were analyzed in this study. The baseline characteristics of all patients and the two groups of β-lactam monotherapy and azithromycin combination therapy are listed in Table 1.

**Table 1:** Characteristics of patients with community-acquired pneumonia.

	All patients n = 1131	β-lactam monotherapy n = 952	Azithromycin combination therapy n = 179	*P* value
Male	795 (70.3)	676 (71.0)	119 (66.5)	0.25
Age (y)	77.0 [69.0–84.0]	78.0 [69.0–84.0]	74.0 [65.0–82.5]	0.001
Smoking status				0.27
Current + Past	726 (64.2)	618 (64.9)	108 (60.3)	
Never	400 (35.4)	330 (34.7)	70 (39.1)	
Unknown	5 (0.4)	4 (0.4)	1 (0.5)	
Comorbidity				
Chronic heart disease	361 (31.9)	293 (30.8)	68 (38.0)	0.07
COPD^†^	284 (25.1)	248 (26.1)	36 (20.1)	0.11
Diabetes mellitus	225 (19.9)	187 (19.6)	38 (21.2)	0.61
Cerebrovascular disease	182 (16.1)	165 (17.3)	17 (9.5)	0.008
Chronic kidney disease	98 (8.7)	71 (7.5)	27 (15.1)	0.002
Malignancy^‡^	90 (8.0)	78 (8.2)	12 (6.7)	0.65
Chronic liver disease	58 (5.1)	45 (4.7)	13 (7.3)	0.19
**Vital signs**				
Body temperature (°C)	37.8 [37.0–38.6]	37.8 [37.0–38.5]	37.8 [37.0–38.7]	0.67
Systolic blood pressure (mmHg)	129 [113–147]	129 [113–147]	128 [112–145]	0.76
Heart rate (beats/min)	97 [84–110]	97 [84–110]	100 [86–113]	0.10
Respiratory rate (breaths/min)	22 [18–25]	21 [18–25]	24 [19–26]	0.30
**Laboratory examinations**				
Albumin (g/dL)	3.2 [2.8–3.6]	3.2 [2.8–3.6]	3.3 [2.9–3.7]	0.005
BUN (mg/dL)	19.0 [14.0–26.0]	19.0 [14.0–25.0]	18.0 [13.0–28.5]	0.93
Creatinine (mg/dL)	0.83 [0.66–1.06]	0.82 [0.66–1.05]	0.84 [0.66–1.14]	0.23
Na (mmol/L)	137 [135–139]	137 [135–139]	137 [135–140]	0.18
Hematocrit (%)	36.7 [33.1–40.0]	36.6 [33.1–40.0]	37.1 [33.5–40.5]	0.25
Platelet (×10^4^/μL)	20.8 [15.5–28.0]	21.2 [15.9–28.6]	18.4 [13.9–24.5]	< 0.001
WBC (×10^3^/μL)	11.4 [8.4–15.2]	11.6 [8.7–15.5]	10.2 [7.5–13.9]	0.005
CRP (mg/L)	116 [52–181]	113 [50–176]	127 [57–212]	0.05
Performance status^§^				0.01
0	278 (24.6)	220 (23.1)	58 (32.4)	
1	584 (51.6)	499 (52.4)	85 (47.5)	
2	217 (19.2)	190 (20.0)	27 (15.1)	
3	30 (2.7)	22 (2.3)	8 (4.5)	
4	22 (1.9)	21 (2.2)	1 (0.6)	
Aspiration pneumonia	280 (24.8)	251 (26.4)	29 (16.2)	0.003
Bacteremia	40 (3.5)	35 (3.7)	5 (2.8)	0.46
Pre-antibiotic therapy	317 (28.0)	267 (28.0)	50 (27.9)	1.00
CURB-65 (score)				0.07
0	106 (9.4)	78 (8.2)	28 (15.6)	
1	356 (31.5)	307 (32.2)	49 (27.4)	
2	426 (37.7)	363 (38.1)	63 (35.2)	
3	200 (17.7)	167 (17.5)	33 (18.4)	
4	40 (3.5)	34 (3.6)	6 (3.4)	
5	3 (0.3)	3 (0.3)	0 (0)	
PSI (score)	96.0 [80.0–118.0]	96.0 [80.0–118.0]	94.0 [76.5–121]	0.28
PSI (class)				0.01
I	15 (1.3)	10 (1.1)	5 (2.8)	
II	143 (12.6)	114 (12.0)	29 (16.2)	
III	318 (28.1)	267 (28.0)	51 (28.5)	
IV	503 (44.5)	440 (46.2)	63 (35.2)	
V	152 (13.4)	121 (12.7)	31 (17.3)	
IDSA/ATS severe criteria				0.57
Yes	284 (25.1)	236 (24.8)	48 (26.8)	
No	847 (74.9)	716 (75.2)	131 (73.2)	
Duration of hospitalization (days)	11.0 [8.0–18.0]	12.0 [8.0–18.0]	10.0 [7.0–19.0]	0.20
In-hospital mortality	62 (5.5)	53 (5.6)	9 (5.0)	0.86
30-day mortality	53 (4.7)	48 (5.0)	5 (2.8)	0.25

Data are presented as median [interquartile range] or n (%).

^†^COPD was diagnosed using the GOLD definition^29^. Patients who were already diagnosed and treated as COPD at other hospitals and had emphysema on chest computed tomography were included.

^‡^This included patients with malignant disease that was active at the time of admission or was diagnosed within 1 y of admission.

^§^The criteria of the Eastern Cooperative Oncology Group were used^30^.

ATS, American Thoracic Society; BUN, blood urea nitrogen; COPD, chronic obstructive pulmonary disease; CRP, C-reactive protein; CURB-65, confusion, urea > 7 mmol/L, respiratory rate ≥30 breaths/min, low blood pressure (systolic <90 mmHg or diastolic ≤60 mmHg), and age ≥65 y; IDSA, Infectious Diseases Society of America; Na, sodium; PSI, Pneumonia Severity Index; WBC, white blood cell.

The specific antimicrobial agents used for β-lactam monotherapy and azithromycin combination therapy are shown in Table 2. In this cohort, two patients who survived after treatment with β-lactam and clarithromycin combination therapy were excluded so that the effect of the addition of azithromycin could be examined alone.

**Table 2:** Antimicrobial agents in β-lactam monotherapy and azithromycin combination therapy.

Antimicrobial agents	β-lactam monotherapy n = 952	Azithromycin combination therapy^†^ n = 179	*P* value
Ampicillin	61 (6.4)	13 (7.3)	0.62
Sulbactam/ampicillin	590 (62.0)	61 (34.1)	<0.001
Tazobactam/piperacillin	41 (4.3)	3 (1.7)	0.14
Ceftriaxone	246 (25.8)	97 (54.2)	<0.001
Cefepime	2 (0.2)	0 (0)	1.0
Cefozopran	1 (0.1)	0 (0)	1.0
Meropenem	11 (1.2)	5 (2.8)	0.16

^†^Oral form at 500 mg/day for 3 days in 18 patients and at 2 g/day single dose in 146 patients; by injection at 500 mg/day in 15 patients.

The characteristics of the patients grouped as having severe or non-severe pneumonia by CURB-65, PSI, and the IDSA/ATS severe pneumonia criteria are presented in Tables S1, S2, and S3, respectively.

### Etiology of pneumonia

The distribution of the causative microorganisms in this study is shown in Table 3. In the β-lactam monotherapy and azithromycin combination therapy groups, atypical pathogens were detected in 20 (2.1%) and 19 patients (10.6%), respectively. No patients were diagnosed with atypical pneumonia by rapid diagnostic tests on admission. If such a diagnosis is given on admission, we usually provide treatment with fluoroquinolones, macrolides, or tetracycline monotherapy. Therefore, no such patients were included in the analysis in the present study.

**Table 3:** Etiology of pneumonia in β-lactam monotherapy and azithromycin combination therapy.

Causative pathogen	All patients	β-lactam monotherapy	Azithromycin combination therapy
	n = 1131 n (%^†^)	n = 952 n (%)	n = 179 n (%)
*Streptococcus pneumoniae*	240 (21.2)	207 (21.7)	33 (18.4)
*Haemophilus influenzae*	91 (8.0)	77 (8.0)	14 (7.8)
*Moraxella catarrhalis*	37 (3.3)	30 (3.2)	7 (3.9)
Methicillin-sensitive *Staphylococcus aureus*	34 (3.0)	31 (3.3)	3 (1.7)
*Streptococcus anginosus* group	27 (2.4)	27 (2.8)	0 (0)
*Streptococcus spp.*	18 (1.6)	17 (1.8)	1 (0.6)
*Klebsiella pneumoniae*	18 (1.6)	15 (1.6)	3 (1.7)
Anaerobes	17 (1.5)	16 (1.7)	1 (0.6)
*Pseudomonas aeruginosa*	15 (1.3)	13 (1.4)	2 (1.1)
*Escherichia coli*	5 (0.4)	4 (0.4)	1 (0.6)
*Corynebacterium spp.*	4 (0.4)	3 (0.3)	1 (0.6)
*Acinetobacter spp.*	2 (0.2)	2 (0.2)	0 (0)
Methicillin-resistant *Staphylococcus aureus*	2 (0.2)	2 (0.2)	0 (0)
Other pathogens^‡^	8 (0.7)	6 (0.6)	2 (1.1)
Atypical pathogens^+^	39 (3.4)	20 (2.1)	19 (10.6)
*Chlamydophila pneumoniae*	23 (2.0)	13 (1.4)	10 (5.6)
*Mycoplasma pneumoniae*	12 (1.1)	7 (0.7)	5 (2.7)
*Legionella pneumophila*	2 (0.2)	0 (0)	2 (1.1)
*Chlamydophila psittaci*	2 (0.2)	0 (0)	2 (1.1)
Unknown	641 (56.7)	535 (56.2)	106 (59.2)

^†^There were 67 patients with multiple etiologies; therefore, the sum of the infection rates is over 100%.

^‡^The other pathogens included influenza virus (2), *Actinomyces spp.* (1), *Citrobacter koseri* (1), *Enterobacter aerogenes* (1), *Kocuria kristinae* (1), *Proteus mirabilis* (1), and *Veillonella spp.* (1).

^§^The atypical pathogens included *Chlamydophila pneumoniae*, *Mycoplasma pneumoniae*, *Legionella pneumophila* and *Chlamydophila psittaci*.

### Outcome

Overall, the 30-day mortality in all patients was 4.7% (53/1131). In addition, 2.8% (25/888) of the patients had a CURB-65 score of 0–2 points, 11.5% (28/243) a CURB-65 score of 3–5 points, 1.3% (6/476) were classified as PSI class I–III, 7.2% (47/655) as PSI class IV–V, 1.2% (16/847) as IDSA/ATS non-severe, and 13.0% (37/284) as IDSA/ATS severe.

Compared with β-lactam monotherapy, azithromycin combination therapy did not significantly reduce the 30-day mortality in all patients (Table 4). When the patients were analyzed according to the severity of CAP (Table 4), azithromycin combination therapy did not significantly improve the prognosis of both the non-severe and severe pneumonia groups by CURB-65 and PSI. However, based on the IDSA/ATS severe criteria, azithromycin combination therapy significantly reduced the 30-day mortality in severe pneumonia (odds ratio [OR] 0.12, 95% confidence interval [CI] 0.007–0.57, *P* = 0.038), but not in non-severe pneumonia (OR 1.85, 95% CI 0.51–5.40, *P* = 0.294). In the β-lactam monotherapy group, the 30-day mortality in patients with atypical pathogens was 10% (2/20), although one of these patients had two causative microorganisms, including *Chlamydophila pneumoniae* and *Haemophilus influenzae*. In the azithromycin combination therapy group, the 30-day mortality in patients with atypical pathogens was 5.3% (1/19).

**Table 4:** The 30-day mortality with β-lactam monotherapy and azithromycin combination therapy in patients with CAP according to severity assessment.

	30-day mortality n (%)		Before IPTW analysis		After IPTW analysis	
	β-lactam monotherapy n = 952	Azithromycin combination therapy n = 179	OR (95% CI)	*P* value	OR (95% CI)	*P* value
All patients n = 1131	48/952 (5.0)	5/179 (2.8)	0.54 (0.19–1.26)	0.198	1.00 (0.34–2.96)	1.0
**CURB-65**						
0–2 n = 888	22/748 (2.9)	3/140 (2.1)	0.72 (0.17–2.12)	0.602	1.73 (0.38–7.80)	0.477
3–5 n = 243	26/204 (12.7)	2/39 (5.1)	0.37 (0.06–1.31)	0.188	0.49 (0.10–2.31)	0.366
**PSI**						
I–IIIn = 476	6/391 (1.5)	0/85 (0)	7.51 × 10^−8^ (NA-4.0 × 10^78^)	0.993	NA	NA
IV–V n = 655	42/561 (7.5)	5/94 (5.3)	0.69 (0.24–1.65)	0.45	0.92 (0.31–2.77)	0.886
**IDSA/ATS severe criteria**						
Non-severe n = 847	12/716 (1.7)	4/131 (3.0)	1.85 (0.51–5.40)	0.294	3.76 (0.94–15.1)	0.062
Severe n = 284	36/236 (15.3)	1/48 (2.0)	0.12 (0.007–0.57)	0.038	0.13 (0.02–0.99)	0.049

ATS, American Thoracic Society; CAP, community-acquired pneumonia; CI, confidence interval; CURB-65, confusion, urea >7 mmol/L, respiratory rate ≥30 breaths/min, low blood pressure (systolic <90 mmHg or diastolic ≤60 mmHg), and age ≥65 y; IDSA, Infectious Diseases Society of America; IPTW, inverse probability of treatment weighting; NA, not available; OR, odds ratio; PSI, Pneumonia Severity Index.

### Outcome after adjustment by inverse probability of treatment weighting (IPTW) analysis

Figures S1–S7 show the standardized mean differences in all patients and in the patients grouped according to CAP severity before and after adjustment by IPTW analysis.

Even after adjusting by IPTW analysis (Table 4), azithromycin combination therapy did not significantly improve the prognosis in all patients or in the patient groups according to the CURB-65 and PSI. Azithromycin combination therapy significantly improved the prognosis of patients classified as severe pneumonia by the IDSA/ATS criteria, but not in non-severe patients.

## Discussion

In the present study, we found that azithromycin combination therapy significantly reduced the 30-day mortality of non-ICU hospitalized CAP patients who satisfied the IDSA/ATS minor criteria for severe pneumonia; however, it did not improve the prognosis of all patients or of the patients grouped as non-severe and severe pneumonia according to the PSI and CURB-65. These results were consistent even after adjusting by IPTW analysis.

A systematic review and meta-analysis by Nie *et al*. in 2014 showed that β-lactam and macrolide dual therapy significantly reduced the mortality of CAP in patients hospitalized in the non-ICU ward, both in patients with mild to moderate pneumonia and in those with severe pneumonia^17^. Thereafter, two RCTs were reported. One RCT by Garin *et al*.^12^ reported no significant differences in mortality, ICU admission, complications, length of stay, and recurrence of pneumonia between β-lactam monotherapy and β-lactam–macrolide combination treatment. Another RCT by Postma *et al*.^13^ reported that empiric β-lactam monotherapy was non-inferior to β-lactam and macrolide combination therapy, with regard to the 90-day mortality in CAP patients hospitalized at non-ICU wards. However, the study by Garin used only clarithromycin as the macrolide and excluded CAP patients who met the PSI class V and IDSA/ATS severe criteria; the study by Postma included many patients with mild to moderate severity. Because of these limitations, the necessity of β-lactam and macrolide combination therapy in severe CAP patients hospitalized at a non-ICU ward is controversial.

Careful selection of hospitalized CAP patients who may benefit from macrolide combination therapy is important to avoid antimicrobial resistance, adverse effects, and the high cost of treatment. A recent systematic review and meta-analysis by Horita *et al*. showed that compared with β-lactam monotherapy, macrolide combination therapy reduced the mortality in severe pneumonia (OR 0.75, 95% CI 0.65–0.86), but not in mild to moderate pneumonia (OR 1.12, 95% CI 0.87–1.45)^14^. However, compared with the present study, that meta-analysis did not show the criteria for severe pneumonia patients who may benefit from macrolide combination therapy.

The superiority of the IDSA/ATS minor criteria over the CURB-65 and PSI in identifying patients who may benefit from azithromycin combination therapy may have been influenced by the differences in each assessment item among the severity scores. In 2016, sepsis was defined as a life-threatening organ dysfunction caused by a dysregulated host response to infection^18^. This report showed that organ dysfunction can be identified as an acute change in the total Sequential Organ Failure Assessment (SOFA) score of ≥2 points due to infection. The SOFA score includes six parameters: PaO_2_/F_I_O_2_ ratio, platelet count, total bilirubin, mean arterial blood pressure, consciousness disturbance, and creatinine level. Among the six parameters of the SOFA score, four items are included in the IDSA/ATS minor criteria (i.e., PaO_2_/F_I_O_2_ ratio ≤250, platelet count <100 × 10^3^/μL, hypotension requiring aggressive fluid resuscitation, and consciousness disturbance); two items are included in the CURB-65 (i.e., low blood pressure and consciousness disturbance); and three items are included in the PSI (PaO_2_ < 60, systolic blood pressure <90, and consciousness disturbance). Among these three pneumonia severity indices, the IDSA/ATS minor criteria include the most items in the SOFA score and PSI was likely to be influenced by the patient’s age and comorbidities. According to these points, the IDSA/ATS minor criteria may better reflect the severity of the infection itself and may therefore be useful in identifying patients who could benefit from azithromycin combination therapy.

The efficacy of macrolide combination therapy for CAP had been proposed to have three mechanisms, including (1) coverage for atypical pathogens, (2) synergistic effect between β-lactams and macrolides, and (3) immunomodulatory effect. However, a meta-analysis of 28 RCTs showed that empiric atypical coverage did not improve the prognosis in hospitalized CAP^19^, and a previous study reported that there was no synergistic effect in ceftriaxone and azithromycin combination therapy^20^. Therefore, although no data from human studies have been reported, the immunomodulatory effect of macrolides seems to be the most important mechanism of efficacy. Macrolides such as azithromycin have immunomodulatory effects on host–pathogen interaction, functions of epithelial and inflammatory cells, improvement of mucociliary clearance, and attenuation of the inflammatory response^21,22^. A recent study by Yoshioka *et al*.^20^ reported that ceftriaxone and azithromycin combination therapy in a mouse model of lethal pneumococcal pneumonia significantly improved the prognosis and suppressed the expressions of CTLA-4 and PD-1 in T helper and T regulatory cells; they suggested that the survival benefits of ceftriaxone and azithromycin combination therapy may be through modulation of immune checkpoints.

This study had some limitations. First, it was conducted at a single center in Japan; therefore, the benefit of azithromycin combination therapy in reducing the mortality of hospitalized patients who satisfied the IDSA/ATS minor criteria for severe CAP should be confirmed by a multicenter RCT. Nevertheless, compared with many previous reports, this study had a prospective observational cohort design, was relatively long-term, included a large number of patients, and excluded possible biases by IPTW analysis. Second, the severe CAP patients who satisfied the IDSA/ATS minor criteria may have been better admitted to the ICU immediately after diagnosis. However, the criteria for ICU admission vary among different facilities and countries, according to the medical circumstances; at our institution, the criteria for ICU admission seemed reasonable. Third, only five patient deaths occurred within 30 days of admission in the azithromycin combination group; therefore, the results should be carefully considered. Fourth, although IPTW analysis can adjust for variables between two groups, we can only adjust the included variables. Therefore, as stated above, whether azithromycin combination therapy reduces the mortality of patients with severe pneumonia should be confirmed by a multicenter RCT.

## Conclusions

β-lactam and azithromycin combination therapy should be considered for non-ICU hospitalized CAP patients who meet the IDSA/ATS minor criteria for severe pneumonia. On the other hand, azithromycin combination therapy might not be necessary for hospitalized patients with mild to moderate CAP. The IDSA/ATS minor criteria for severe pneumonia might be useful in identifying the patients who would benefit from azithromycin combination therapy, but an RCT is needed to confirm this finding.

## Methods

### Study design and setting

This prospective, observational, cohort study enrolled consecutive patients with CAP hospitalized in the non-ICU ward of Kurashiki Central Hospital between October 2010 and November 2016. CAP was diagnosed based on the IDSA/ATS guidelines^1^ as the presence of at least one of the clinical symptoms of cough, sputum, fever, dyspnea, and pleuritic chest pain, plus at least one finding of coarse crackles on auscultation or elevated inflammatory biomarkers, in addition to a new infiltrate on chest radiography. The exclusion criteria were age <15 y, ICU transfer on admission, β-lactam antibiotics and azithromycin not used as initial treatment, hospital-acquired pneumonia (caused more than 48 h from admission), and healthcare-associated pneumonia^23^. The criteria for healthcare-associated pneumonia are as follows: (1) hospitalization for ≥2 days in the preceding 90 days; (2) residence in a nursing home or extended care facility; (3) receiving infusion therapy including antibiotics; (4) receiving outpatient hemodialysis or peritoneal dialysis within 30 days before admission; and (5) home wound care.

This study was performed as a clinical study for pneumonia (UMIN000004353) and was approved by the institutional review board of Kurashiki Central Hospital (approval number 641). This study was also conducted in accordance with the amended Declaration of Helsinki. Based on the Ethical Guidelines for Medical and Health Research Involving Human Subjects of the Ministry of Health, Labour and Welfare, the research participants were notified or the public was made aware of information concerning the research on the Internet. All patients gave their informed consent to participate in this study by being given opportunities to refuse to participate (opt-out system).

In all patients, the severity of pneumonia was assessed on admission with the use of the CURB-65 score [confusion, urea >7 mmol/L, respiratory rate ≥30 breaths/min, low blood pressure (systolic <90 mmHg or diastolic ≤60 mmHg), and age ≥65 y]^15^, PSI^16^, and IDSA/ATS severe pneumonia criteria^1^. Patients who meet the major criteria of the IDSA/ATS for severe pneumonia (i.e., mechanical ventilation with endotracheal intubation and/or septic shock requiring vasopressors) are usually treated in the ICU; therefore, in this study, we adapted the minor criteria. Patients who fulfilled at least three minor criteria were classified as having severe pneumonia^1^. We also defined a CURB-65 score of 3–5 points and PSI classes IV and V as severe pneumonia, and a CURB-65 score of 0–2 points and PSI classes I–III are non-severe pneumonia, in accordance with previous reports^1,15,24,25^.

All patients received antimicrobial agents based on the decision of the attending physician and according to the CAP guidelines of the Japanese Respiratory Society^4^. We typically use β-lactam antibiotics, such as a β-lactam/β-lactamase inhibitor combination or cephalosporin, as the initial treatment for patients with CAP hospitalized in the non-ICU ward^26^. We may use β-lactam and macrolide combination therapy if the patients are clinically suspected to have been infected by atypical pathogens. In our hospital, patients who needed mechanical ventilation and/or vasopressor drugs were basically treated in the ICU. Patients with severe hypoxemia and/or shock who did not need mechanical ventilation and vasopressor were also treated in the ICU, depending on the discretion of the attending physician.

### Microbiologic examination

To detect the causative microorganisms of CAP, we examined sputum and blood cultures and collected blood to measure serum antibodies on admission. A bacterial cause was identified if the following criteria were met: (1) positive sputum culture of more than 1+ on a qualitative test or 10^5^ on a quantitative test, with significant Gram stain; (2) positive blood culture, excluding bacterial contaminants; (3) positive pleural fluid culture; (4) positive urinary antigen test for *Streptococcus pneumoniae* and *Legionella pneumophila*; (5) seroconversion or a four-fold increase in the antibodies for *Mycoplasma pneumoniae* and *C. pneumoniae*; and (6) ≥1:320 on a single particle agglutination antibody test for *M. pneumonia* (FUJIREBIO; Tokyo, Japan) or ≥2.0 cutoff index on a *C. pneumoniae* IgM antibody test (Hitazyme^®^ assay; Hitachi Chemical, Tokyo, Japan).

### Outcome

The primary outcome was 30-day in-hospital mortality. We checked all the patients’ charts after 30 days from discharge who were discharged alive within 30 days from admission to see whether they had died or been readmitted.

### Statistical analysis

Continuous variables were expressed as median and interquartile range, and categorical variables were expressed as counts (percentage). Continuous variables were analyzed using a non-parametric Mann–Whitney *U*-test, and categorical variables were assessed using Fisher’s exact test. We analyzed whether β-lactam and azithromycin combination therapy (azithromycin combination therapy), in comparison with β-lactam monotherapy, improved the prognosis of mild to severe pneumonia in all patients and in patients grouped according to the two severity classes (i.e., non-severe and severe) based on the existing severity scoring systems (CURB-65, PSI, and IDSA/ATS severe criteria). For comparison of the 30-day mortality between β-lactam monotherapy and azithromycin combination therapy, we used propensity score (PS) methods to reduce biases and the influence of the patients’ characteristics, such as age, comorbidities, and vital signs, laboratory examinations, and pneumonia severity, on the effects of treatment on outcome. Among the four PS methods, stratification, matching, weighting, and covariate adjustment^27^, IPTW was selected for analysis because it has been reported to result in a lower mean squared error when estimating treatment effects^28^. The PS was estimated by multivariate logistic regression analysis involving 15 covariates: age, sex, chronic obstructive pulmonary disease, malignancy, performance status, aspiration pneumonia, systolic blood pressure, respiratory rate, C-reactive protein, albumin, blood urea nitrogen, platelet, PSI score, CURB-65 class, and IDSA/ATS severe pneumonia classification. We selected these 15 variables because they were significantly different between the two treatment groups and have been reported to be prognostic factors^15,16,26^ that can influence the selection of therapy, although not significantly. We dealt with incorrect standard errors using robust standard errors, and used the R package ‘sandwich’ (version 2.5-0; Vienna, Austria). All statistical tests were two-tailed, a *P* value of <0.05 was considered significant. Analyses were performed using R (version 3.0.3).

## Supplementary information

Supplementary information

## Data Availability

The data sets used and analyzed during the current study are available from the corresponding author upon reasonable request.
