# Supplementary material for: Azithromycin combination therapy for community-acquired pneumonia: propensity score analysis
Source: Sci Rep. 2019 Dec 5;9:18406. doi: 10.1038/s41598-019-54922-4 (PMC6895050; doi:10.1038/s41598-019-54922-4)

**Title: Azithromycin combination therapy for community-acquired pneumonia: propensity score analysis**

Akihiro Ito^1*^, Tadashi Ishida^1^, Hiromasa Tachibana^1,2^, Hironobu Tokumasu^3^, Akio Yamazaki^1,4^, Yasuyoshi Washio^1,5^

**Table S1.** Comparison of monotherapy with combination therapy in patients with CAP according to the CURB-65 score.

|  | CURB-65 | | | | | |
| --- | --- | --- | --- | --- | --- | --- |
|  | 0–2  n = 888 | |  | 3–5  n = 243 | |  |
|  | β-lactam  monotherapy  n = 748 | Azithromycin combination  therapy  n = 140 | *P* value | β-lactam  monotherapy  n = 204 | Azithromycin combination  therapy  n = 39 | *P* value |
| Male | 531 (71.0) | 91 (65.0) | 0.19 | 145 (71.1) | 28 (71.8) | 1.00 |
| Age (y) | 76  [68–83] | 73  [63–81] | 0.004 | 82  [77–86] | 79  [72–84] | 0.03 |
| Smoking status |  |  | 0.61 |  |  | 0.26 |
| Current + Past | 487 (65.1) | 87 (62.1) |  | 131 (64.2) | 21 (53.8) |  |
| Never | 259 (34.6) | 52 (37.2) |  | 71 (34.8) | 18 (46.2) |  |
| Unknown | 2 (0.3) | 1 (0.7) |  | 2 (1.0) | 0 (0) |  |
| Comorbidity |  |  |  |  |  |  |
| Chronic heart disease | 191 (25.5) | 44 (31.4) | 0.18 | 102 (50.0) | 24 (61.5) | 0.25 |
| COPD^†^ | 197 (26.3) | 29 (20.7) | 0.19 | 51 (25.0) | 7 (17.9) | 0.46 |
| Diabetes mellitus | 142 (19.0) | 25 (17.9) | 0.85 | 45 (22.1) | 13 (33.3) | 0.19 |
| Cerebrovascular disease | 114 (15.2) | 12 (8.6) | 0.05 | 51 (25.0) | 5 (12.8) | 0.15 |
| Chronic kidney disease | 49 (6.6) | 18 (12.9) | 0.02 | 22 (10.8) | 9 (23.1) | 0.06 |
| Malignancy^‡^ | 56 (7.5) | 11 (7.9) | 1.00 | 22 (10.8) | 1 (2.6) | 0.19 |
| Chronic liver disease | 38 (5.1) | 11 (7.9) | 0.26 | 7 (3.4) | 2 (5.1) | 0.96 |
| Vital signs |  |  |  |  |  |  |
| Body temperature (°C) | 37.8  [37.0–38.5] | 37.9  [37.0–38.7] | 0.43 | 37.7  [36.9–38.6] | 37.6  [37.1–38.3] | 0.61 |
| Systolic blood pressure (mmHg) | 132  [117–148] | 132  [117.8–147.3] | 0.97 | 118  [100–137] | 112  [103–135] | 0.67 |
| Heart rate (beats/min) | 97  [85–110] | 100  [89–113] | 0.03 | 96  [81–110] | 91  [82–111] | 0.72 |
| Respiratory rate (breaths/min) | 20  [18–24] | 22  [18–24] | 0.48 | 24  [20–30] | 25  [21–31] | 0.41 |
| Laboratory examinations |  |  |  |  |  |  |
| Albumin (g/dL) | 3.2  [2.8–3.6] | 3.4  [2.9–3.8] | 0.002 | 3.2  [2.7–3.5] | 3.2  [2.8–3.5] | 0.94 |
| BUN (mg/dL) | 17  [13–22] | 16  [12–21] | 0.25 | 27  [22–37] | 33  [22–45] | 0.09 |
| Creatinine (mg/dL) | 0.79  [0.63–1.00] | 0.80  [0.64–1.00] | 0.58 | 1.00  [0.81–1.37] | 1.2  [0.86–1.72] | 0.09 |
| Na (mmol/L) | 137  [135–139] | 138  [135–140] | 0.06 | 138  [135–140] | 137  [136–139] | 0.47 |
| Hematocrit (%) | 37.0  [33.4–40.2] | 37.8  [33.9–41.0] | 0.16 | 35.6  [31.5–39.2] | 35.3  [31.9–38.1] | 0.93 |
| Platelet (×10^4^/μL) | 22.1  [16.1–29.7] | 19.1  [14.1–25.2] | 0.002 | 20.2  [14.8–24.9] | 16.7  [12.8–21.8] | 0.02 |
| WBC (×10^3^/μL) | 11.6  [8.8–15.4] | 9.7  [7.2–13.2] | 0.004 | 11.3  [8.2–15.9] | 12.6  [8.6–15.5] | 0.70 |
| CRP (mg/L) | 113  [51–177] | 123  [52–189] | 0.68 | 121  [44–168] | 165  [100–247] | <0.001 |
| Performance status^§^ |  |  | 0.09 |  |  | 0.20 |
| 0 | 196 (26.2) | 49 (35.0) |  | 24 (11.8) | 9 (23.1) |  |
| 1 | 395 (52.8) | 67 (47.9) |  | 104 (51.0) | 18 (46.2) |  |
| 2 | 128 (17.1) | 17 (12.1) |  | 62 (30.4) | 10 (25.6) |  |
| 3 | 17 (2.3) | 6 (4.3) |  | 5 (2.4) | 2 (5.1) |  |
| 4 | 12 (1.6) | 1 (0.7) |  | 9 (4.4) | 0 (0) |  |
| Aspiration pneumonia | 164 (21.9) | 15 (10.7) | 0.004 | 87 (42.6) | 14 (35.9) | 0.54 |
| Bacteremia | 24 (5.1) | 2 (2.0) | 0.28 | 11 (8.0) | 3 (8.3) | 1.00 |
| PSI (score) | 90  [77–107] | 87  [73–106] | 0.23 | 125  [110–144] | 127  [101–138] | 0.70 |
| PSI (class) |  |  | 0.004 |  |  | 0.01 |
| I | 10 (1.3) | 5 (3.6) |  | 0 (0) | 0 (0) |  |
| II | 114 (15.2) | 27 (19.3) |  | 0 (0) | 2 (5.1) |  |
| III | 253 (33.8) | 48 (34.3) |  | 14 (6.9) | 3 (7.7) |  |
| IV | 329 (44.0) | 44 (31.4) |  | 111 (54.4) | 19 (48.7) |  |
| V | 42 (5.6) | 16 (11.4) |  | 79 (38.7) | 15 (38.5) |  |
| IDSA/ATS severe criteria |  |  | 0.87 |  |  | 0.68 |
| Yes | 105 (14.0) | 21 (15.0) |  | 131 (64.2) | 27 (69.2) |  |
| No | 643 (86.0) | 119 (85.0) |  | 73 (35.8) | 12 (30.8) |  |

Data are presented as median [interquartile range] or n (%)

^†^COPD was diagnosed using the GOLD definition^29^. Patients who were already diagnosed and treated as COPD at other hospitals and had emphysema on chest computed tomography were included.

^‡^This included patients with malignant disease that was active at the time of admission or was diagnosed within 1 y of admission.

^§^The criteria of the Eastern Cooperative Oncology Group were used^30^.

ATS, American Thoracic Society; BUN, blood urea nitrogen; CAP, community-acquired pneumonia; COPD, chronic obstructive pulmonary disease; CRP, C-reactive protein; CURB-65, confusion, urea >7 mmol/L, respiratory rate ≥30 breaths/min, low blood pressure (systolic <90 mmHg or diastolic ≤60 mmHg), and age ≥65 y; IDSA, Infectious Diseases Society of America; Na, sodium; PSI, Pneumonia Severity Index; WBC, white blood cell.

**Table S2.** Comparison of monotherapy with combination therapy in patients with CAP according to the PSI score.

|  | PSI | | | | | |
| --- | --- | --- | --- | --- | --- | --- |
|  | I–III  n = 476 | |  | IV–V  n = 655 | |  |
|  | β-lactam  monotherapy  n = 391 | Azithromycin combination  therapy  n = 85 | *P* value | β-lactam  monotherapy  n = 561 | Azithromycin combination  therapy  n = 94 | *P* value |
| Male | 243 (62.1) | 45 (52.9) | 0.15 | 433 (77.2) | 74 (78.7) | 0.84 |
| Age (y) | 71  [63–79] | 67  [53–75] | 0.003 | 81  [75–86] | 80  [72–85] | 0.13 |
| Smoking status |  |  | 0.03 |  |  | 0.53 |
| Current + Past | 242 (61.9) | 41 (48.2) |  | 376 (67.0) | 67 (71.3) |  |
| Never | 148 (37.8) | 43 (50.6) |  | 182 (32.4) | 27 (28.7) |  |
| Unknown | 1 (0.3) | 1 (1.2) |  | 3 (0.5) | 0 (0) |  |
| Comorbidity |  |  |  |  |  |  |
| Chronic heart disease | 74 (18.9) | 17 (20.0) | 0.94 | 219 (39.0) | 51 (54.3) | 0.008 |
| COPD^†^ | 88 (22.5) | 9 (10.6) | 0.02 | 160 (28.5) | 27 (28.7) | 1.0 |
| Diabetes mellitus | 61 (15.6) | 12 (14.1) | 0.86 | 126 (22.5) | 26 (27.7) | 0.33 |
| Cerebrovascular disease | 27 (6.9) | 5 (5.9) | 0.92 | 138 (24.6) | 12 (12.8) | 0.02 |
| Chronic kidney disease | 10 (2.6) | 8 (9.4) | 0.007 | 61 (10.9) | 19 (20.2) | 0.02 |
| Malignancy^‡^ | 8 (2.0) | 2 (2.4) | 1.00 | 70 (12.5) | 10 (10.6) | 0.74 |
| Chronic liver disease | 9 (2.3) | 5 (5.9) | 0.16 | 36 (6.4) | 8 (8.5) | 0.60 |
| Vital signs |  |  |  |  |  |  |
| Body temperature (°C) | 37.8  [37.1–38.5] | 38.0  [37.4–39.0] | 0.03 | 37.7  [36.9–38.6] | 37.6  [36.7–38.5] | 0.12 |
| Systolic blood pressure (mmHg) | 130  [115–146] | 130  [115–145] | 0.86 | 129  [111–149] | 125  [109–146] | 0.52 |
| Heart rate (beats/min) | 98  [85–111] | 100  [87–111] | 0.24 | 96  [84–109] | 98  [83–113] | 0.27 |
| Respiratory rate (breaths/min) | 20  [18–24] | 20  [18–24] | 0.25 | 23  [20–28] | 24  [20–28] | 0.40 |
| Laboratory examinations |  |  |  |  |  |  |
| Albumin (g/dL) | 3.3  [2.9–3.6] | 3.4  [3.0–3.9] | 0.002 | 3.2  [2.7–3.6] | 3.2  [2.7–3.6] | 0.47 |
| BUN (mg/dL) | 15  [12–19] | 14  [11–17] | 0.02 | 22  [16–31] | 28  [18–40] | 0.002 |
| Creatinine (mg/dL) | 0.75  [0.60–0.90] | 0.74  [0.61–0.91] | 0.99 | 0.93  [0.70–1.22] | 1.0  [0.79–1.52] | 0.01 |
| Na (mmol/L) | 137  [135–139] | 137  [134–140] | 0.58 | 137  [134–140] | 137  [136–140] | 0.20 |
| Hematocrit (%) | 37.6  [34.7–40.7] | 37.9  [34.8–41.8] | 0.50 | 35.7  [31.7–39.4] | 36.3  [32.4–39.8] | 0.50 |
| Platelet (×10^4^/μL) | 23.4  [18.1–31.1] | 21.3  [15.4–28.7] | 0.01 | 20.2  [14.8–26.3] | 16.8  [13.0–21.9] | <0.001 |
| WBC (×10^3^/μL) | 12.0  [9.3–15.5] | 10.0  [7.3–13.3] | 0.001 | 11.2  [8.4–15.3] | 10.2  [7.6–14.3] | 0.24 |
| CRP (mg/L) | 118  [55–183] | 109  [51–174] | 0.36 | 111  [48–170] | 148  [77–232] | <0.001 |
| Performance status^§^ |  |  | 0.22 |  |  | 0.11 |
| 0 | 142 (36.3) | 42 (49.4) |  | 78 (13.9) | 16 (17.0) |  |
| 1 | 196 (50.1) | 34 (40.0) |  | 303 (54.0) | 51 (54.3) |  |
| 2 | 47 (12.0) | 7 (8.2) |  | 143 (25.5) | 20 (21.3) |  |
| 3 | 3 (0.8) | 1 (1.2) |  | 19 (3.4) | 7 (7.4) |  |
| 4 | 3 (0.8) | 1 (1.2) |  | 18 (3.2) | 0 (0) |  |
| Aspiration pneumonia | 39 (10.0) | 4 (4.7) | 0.18 | 212 (37.8) | 25 (26.6) | 0.048 |
| Bacteremia | 12 (5.2) | 2 (3.6) | 0.88 | 23 (6.1) | 3 (3.8) | 0.58 |
| PSI (score) | 77  [69–84] | 75  [65–83] | 0.24 | 114  [101–128] | 121  [100–135] | 0.16 |
| CURB-65 (score) |  |  | 0.04 |  |  | 0.91 |
| 0 | 74 (18.9) | 27 (31.8) |  | 4 (0.7) | 1 (1.1) |  |
| 1 | 189 (48.3) | 33 (38.8) |  | 118 (21.0) | 16 (17.0) |  |
| 2 | 114 (29.2) | 20 (23.5) |  | 249 (44.4) | 43 (45.7) |  |
| 3 | 14 (3.6) | 5 (5.9) |  | 153 (27.3) | 28 (29.8) |  |
| 4 | 0 (0) | 0 (0) |  | 34 (6.1) | 6 (6.4) |  |
| 5 | 0 (0) | 0 (0) |  | 3 (0.5) | 0 (0) |  |
| IDSA/ATS severe criteria |  |  | 0.87 |  |  | 0.26 |
| Yes | 23 (5.9) | 6 (7.1) |  | 213 (38.0) | 42 (44.7) |  |
| No | 368 (94.1) | 79 (92.9) |  | 348 (62.0) | 52 (55.3) |  |

Data are presented as median [interquartile range] or n (%)

^†^COPD was diagnosed using the GOLD definition^29^. Patients who were already diagnosed and treated as COPD at other hospitals and had emphysema on chest computed tomography were included.

^‡^This included patients with malignant disease that was active at the time of admission or was diagnosed within 1 y of admission.

^§^The criteria of the Eastern Cooperative Oncology Group were used^30^.

ATS, American Thoracic Society; BUN, blood urea nitrogen; CAP, community-acquired pneumonia; COPD, chronic obstructive pulmonary disease; CRP, C-reactive protein; CURB-65, confusion, urea >7 mmol/L, respiratory rate ≥30 breaths/min, low blood pressure (systolic <90 mmHg or diastolic ≤60 mmHg), and age ≥65 y; IDSA, Infectious Diseases Society of America; Na, sodium; PSI, Pneumonia Severity Index; WBC, white blood cell.

**Table S3.** Comparison of monotherapy with combination therapy in patients with CAP according to the IDSA/ATS criteria.

|  | IDSA/ATS severe criteria | | | | | |
| --- | --- | --- | --- | --- | --- | --- |
|  | Non-severe  n = 847 | |  | Severe  n = 284 | |  |
|  | β-lactam  monotherapy  n = 716 | Azithromycin combination  therapy  n = 131 | *P* value | β-lactam  monotherapy  n = 236 | Azithromycin combination  therapy  n = 48 | *P* value |
| Male | 502 (70.1) | 84 (64.1) | 0.21 | 174 (73.7) | 35 (72.9) | 1.0 |
| Age (y) | 77  [69–83] | 73  [63–82] | 0.003 | 80  [73–86] | 76  [71–84] | 0.04 |
| Smoking status |  |  | 0.22 |  |  | 1.0 |
| Current + Past | 460 (64.2) | 76 (58.0) |  | 158 (67.0) | 32 (66.7) |  |
| Never | 253 (35.3) | 54 (41.2) |  | 77 (32.6) | 16 (33.3) |  |
| Unknown | 3 (0.4) | 1 (0.8) |  | 1 (0.4) | 0 (0) |  |
| Comorbidity |  |  |  |  |  |  |
| Chronic heart disease | 194 (27.1) | 40 (30.5) | 0.48 | 99 (41.9) | 28 (58.3) | 0.05 |
| COPD^†^ | 181 (25.3) | 26 (19.8) | 0.22 | 67 (28.4) | 10 (20.8) | 0.37 |
| Diabetes mellitus | 125 (17.5) | 21 (16.0) | 0.79 | 62 (26.3) | 17 (35.4) | 0.27 |
| Cerebrovascular disease | 117 (16.3) | 14 (10.7) | 0.13 | 48 (20.3) | 3 (6.3) | 0.03 |
| Chronic kidney disease | 43 (6.0) | 15 (11.5) | 0.04 | 28 (11.9) | 12 (25.0) | 0.03 |
| Malignancy^‡^ | 54 (7.5) | 7 (5.3) | 0.48 | 24 (10.2) | 5 (10.4) | 1.0 |
| Chronic liver disease | 30 (4.2) | 11 (8.4) | 0.07 | 15 (6.4) | 2 (4.2) | 0.80 |
| Vital signs |  |  |  |  |  |  |
| Body temperature (°C) | 37.8  [37.0–38.5] | 37.9  [37.1–38.8] | 0.52 | 37.7  [36.9–38.6] | 37.7  [37.0–38.4] | 0.89 |
| Systolic blood pressure (mmHg) | 130  [114–147] | 128  [114–145] | 0.55 | 125  [107–149] | 129  [109–145] | 0.70 |
| Heart rate (beats/min) | 96  [84–109] | 98  [85–112] | 0.22 | 99  [86–112] | 104  [90–114] | 0.29 |
| Respiratory rate (breaths/min) | 20  [18–24] | 20  [18–24] | 0.39 | 24  [21–30] | 25  [22–30] | 0.82 |
| Laboratory examinations |  |  |  |  |  |  |
| Albumin (g/dL) | 3.2  [2.8–3.6] | 3.4  [3.0–3.8] | 0.002 | 3.1  [2.7–3.5] | 3.2  [2.7–3.6] | 0.66 |
| BUN (mg/dL) | 17  [13–23] | 16  [12–21] | 0.14 | 25  [20–37] | 29  [21–44] | 0.17 |
| Creatinine (mg/dL) | 0.79  [0.64–1.00] | 0.82  [0.64–1.00] | 0.56 | 0.98  [0.75–1.35] | 1.14  [0.77–1.60] | 0.18 |
| Na (mmol/L) | 137  [135–139] | 137  [135–140] | 0.11 | 137  [133–140] | 137  [135–140] | 0.96 |
| Hematocrit (%) | 36.8  [33.4–39.9] | 37.5  [34.1–41.2] | 0.08 | 36.0  [32.3–40.0] | 35.2  [31.8–39.4] | 0.58 |
| Platelet (×10^4^/μL) | 22.3  [16.7–29.7] | 19.6  [15.2–25.4] | 0.004 | 19.1  [14.3–24.6] | 15.3  [10.8–21.8] | 0.009 |
| WBC (×10^3^/μL) | 11.7  [8.9–15.4] | 10.0  [7.4–13.4] | 0.002 | 10.9  [7.8–15.7] | 10.4  [7.8–15.8] | 0.63 |
| CRP (mg/L) | 110  [48–174] | 123  [55–182] | 0.45 | 121  [56–178] | 162  [79–257] | 0.01 |
| Performance status^§^ |  |  | 0.002 |  |  | 0.55 |
| 0 | 190 (26.5) | 42 (49.4) |  | 30 (12.7) | 7 (14.6) |  |
| 1 | 373 (52.1) | 34 (40.0) |  | 126 (53.4) | 28 (58.3) |  |
| 2 | 131 (18.3) | 7 (8.2) |  | 59 (25.0) | 12 (25.0) |  |
| 3 | 13 (1.8) | 1 (1.2) |  | 9 (3.8) | 1 (2.1) |  |
| 4 | 9 (1.3) | 1 (1.2) |  | 12 (5.1) | 0 (0) |  |
| Aspiration pneumonia | 148 (20.7) | 15 (11.5) | 0.02 | 103 (43.6) | 14 (29.2) | 0.09 |
| Bacteremia | 22 (4.9) | 0 (0) | 0.06 | 13 (7.8) | 5 (11.1) | 0.68 |
| CURB-65 (score) |  |  | 0.02 |  |  | 0.90 |
| 0 | 76 (10.6) | 28 (21.4) |  | 2 (0.9) | 0 (0) |  |
| 1 | 289 (40.4) | 46 (35.1) |  | 18 (7.6) | 3 (6.3) |  |
| 2 | 278 (38.8) | 45 (34.4) |  | 85 (36.0) | 18 (37.5) |  |
| 3 | 70 (9.8) | 11 (8.4) |  | 97 (41.1) | 22 (45.8) |  |
| 4 | 3 (0.4) | 1 (0.8) |  | 31 (13.1) | 5 (10.4) |  |
| 5 | 0 (0) | 0 (0) |  | 3 (1.3) | 0 (0) |  |
| PSI (score) | 90  [77–105] | 85  [73–101] | 0.09 | 125  [108–145] | 126  [100–139] | 0.71 |
| PSI (class) |  |  | 0.003 |  |  | 0.17 |
| I | 10 (1.4) | 5 (3.8) |  | 0 (0) | 0 (0) |  |
| II | 111 (15.5) | 26 (19.9) |  | 3 (1.3) | 3 (6.3) |  |
| III | 247 (34.5) | 48 (36.6) |  | 20 (8.5) | 3 (6.3) |  |
| IV | 319 (44.6) | 40 (30.5) |  | 121 (51.3) | 23 (47.9) |  |
| V | 29 (4.1) | 12 (9.2) |  | 92 (39.0) | 19 (39.6) |  |

Data are presented as median [interquartile range] or n (%)

^†^COPD was diagnosed using the GOLD definition^29^. Patients who were already diagnosed and treated as COPD at other hospitals and had emphysema on chest computed tomography were included.

^‡^This included patients with malignant disease that was active at the time of admission or was diagnosed within 1 y of admission.

^§^The criteria of the Eastern Cooperative Oncology Group were used^30^.

ATS, American Thoracic Society; BUN, blood urea nitrogen; CAP, community-acquired pneumonia; COPD, chronic obstructive pulmonary disease; CRP, C-reactive protein; CURB-65, confusion, urea >7 mmol/L, respiratory rate ≥30 breaths/min, low blood pressure (systolic <90 mmHg or diastolic ≤60 mmHg), and age ≥65 y; IDSA, Infectious Diseases Society of America; Na, sodium; PSI, Pneumonia Severity Index; WBC, white blood cell.

**Figure S1.** The standardized mean differences (SMDs) of all covariates were plotted before and after IPTW adjustment in all patients. After IPTW analyses, the SMDs were less than 0.1 in all covariates.


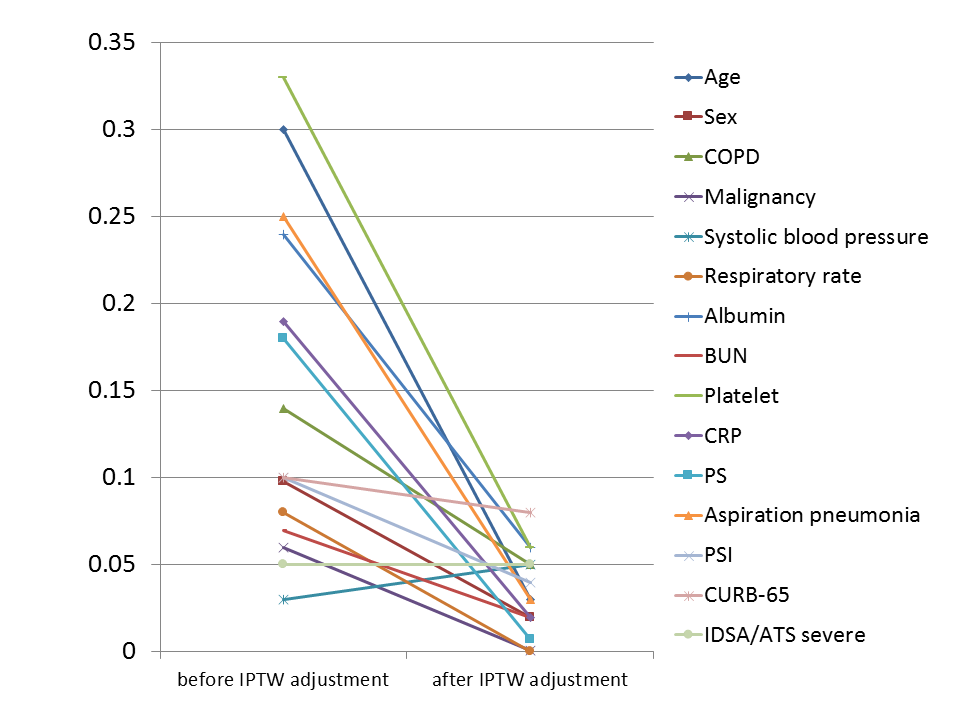


**Figure S2.** The SMDs of all covariates were plotted before and after IPTW adjustment in those with CURB-65 0–2 points. After IPTW analyses, the SMDs were less than 0.1 in all covariates.


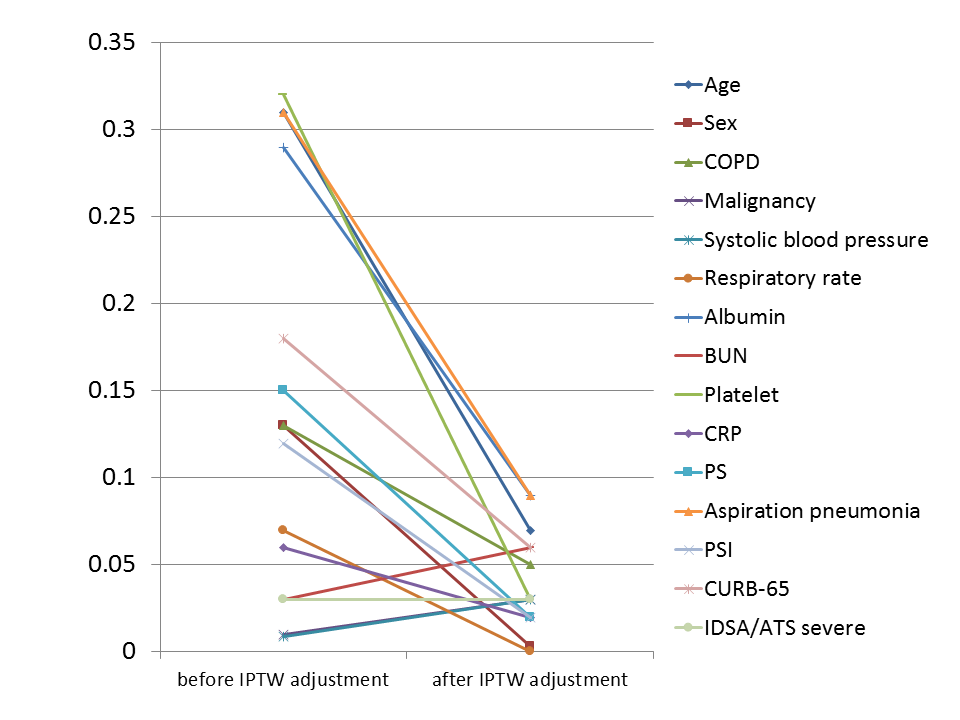


**Figure S3.** The SMDs of all covariates were plotted before and after IPTW adjustment in CURB-65 3–5 points. After IPTW analyses, the SMDs were less than 0.1 in all covariates, except for sex (SMD = 0.23), COPD (SMD = 0.12), respiratory rate (SMD = 0.14), and IDSA/ATS severe criteria (SMD = 0.11).


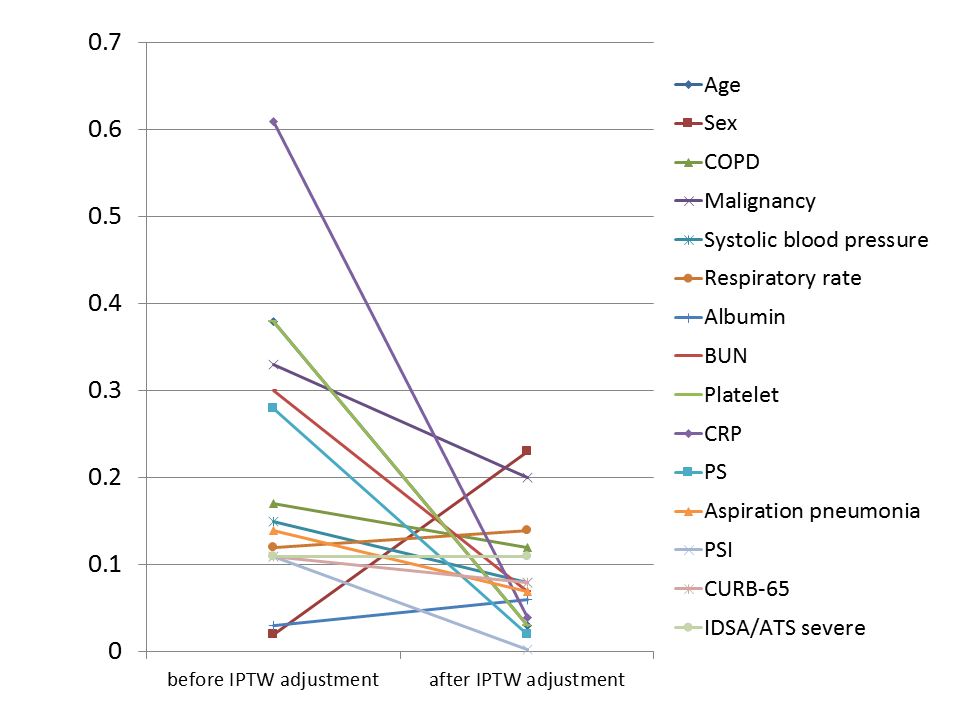


**Figure S4.** The SMDs of all covariates were plotted before and after IPTW adjustment in PSI class I–III. After IPTW analyses, the SMDs were less than 0.1 in all covariates except for COPD (SMD = 0.12) and PS (SMD = 0. 28).


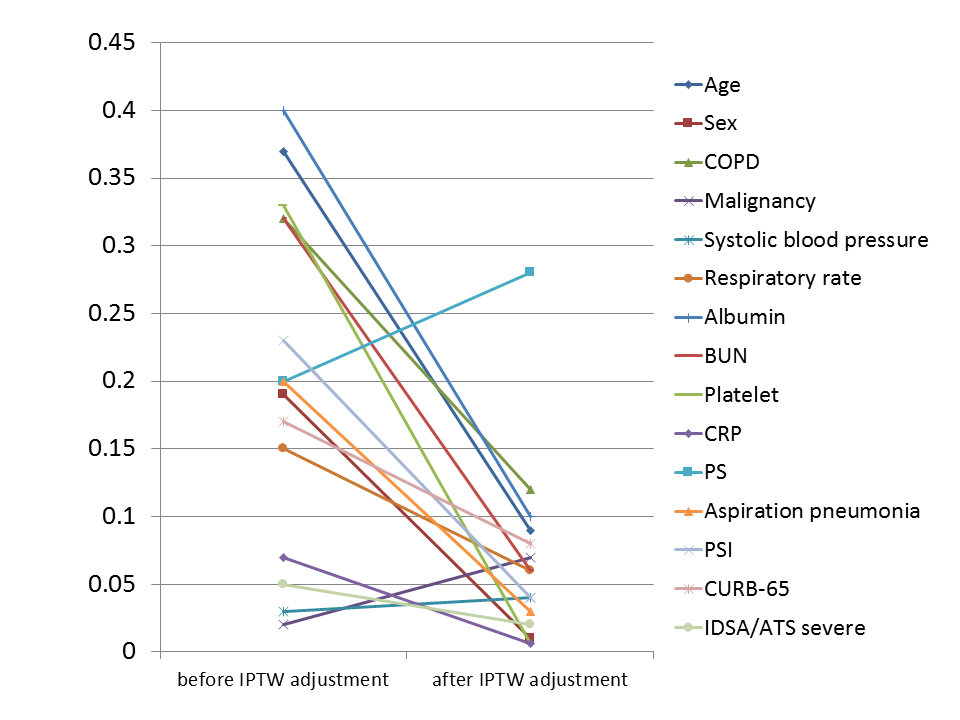


**Figure S5.** The SMDs of all covariates were plotted before and after IPTW adjustment in PSI class IV–V. After IPTW analyses, the SMDs were less than 0.1 in all covariates except for platelet (SMD = 0.15), PS (SMD = 0.12), CURB-65 (SMD = 0.15), and PSI (SMD = 0.11).


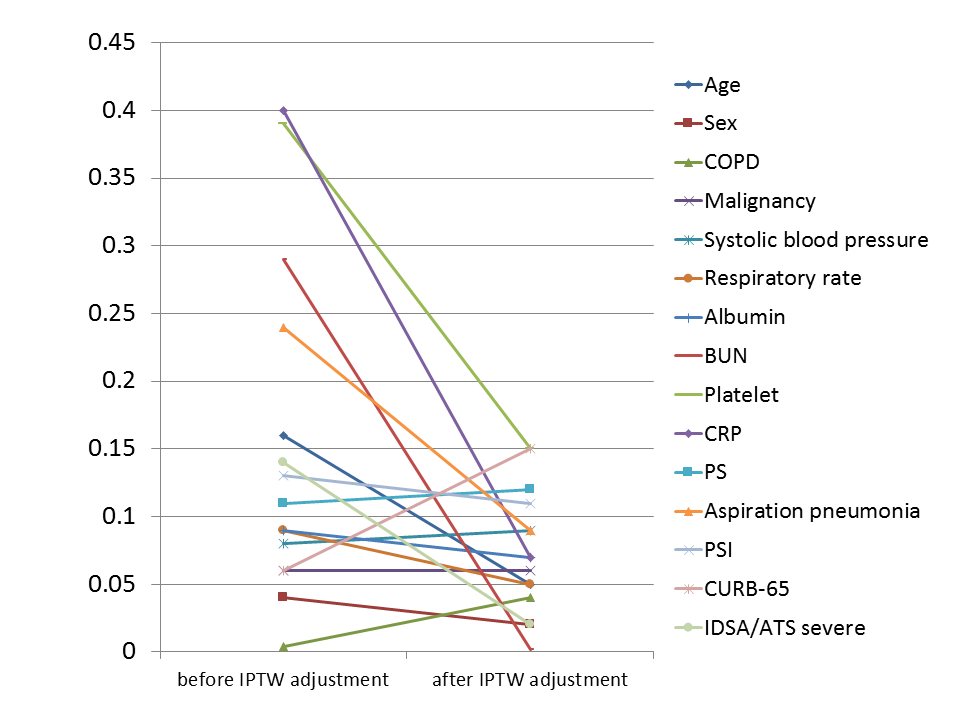


**Figure S6.** The SMDs of all covariates were plotted before and after IPTW adjustment in those classified as IDSA/ATS non-severe. After IPTW analyses, the SMDs were less than 0.1 in all covariates.


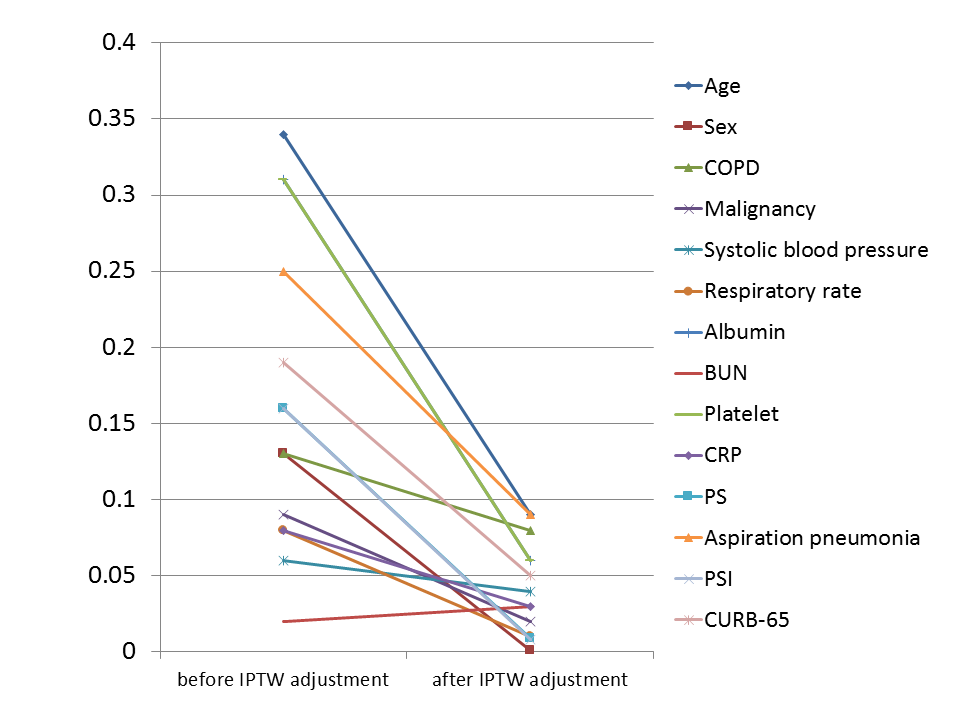


**Figure S7.** The SMDs of all covariates were plotted before and after IPTW adjustment in those classified as IDSA/ATS severe. After IPTW analyses, the SMDs were less than 0.1 in all covariates except for age (SMD = 0.13), systolic blood pressure (SMD = 0.12), and BUN (SMD = 0.11).


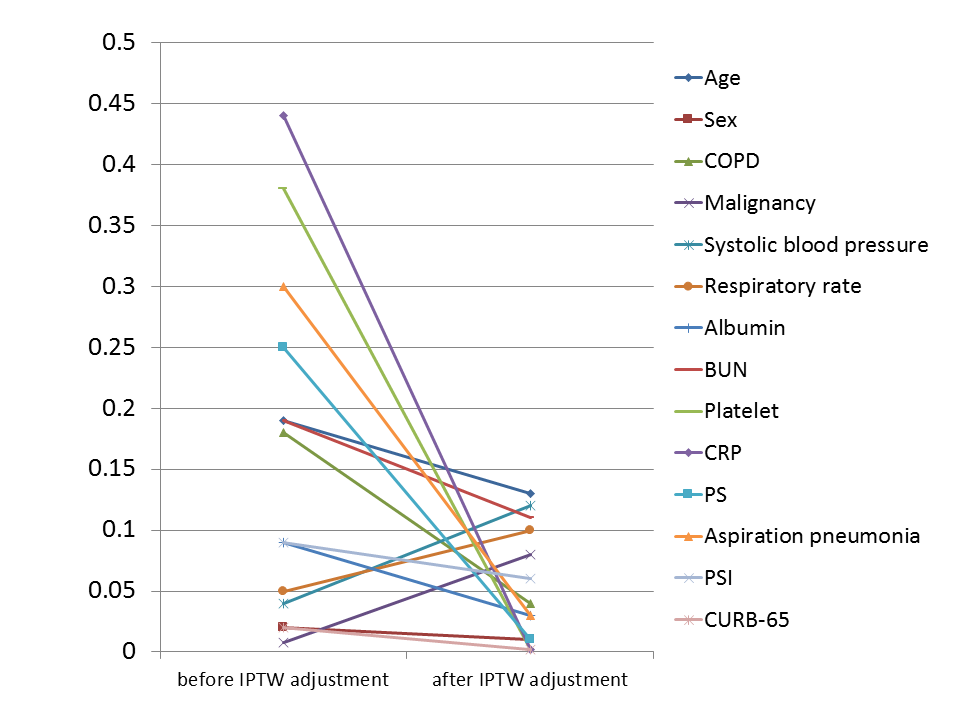

Supplement: Supplementary file 1 — Supplementary information [file 41598_2019_54922_MOESM1_ESM.docx]
